# A Holocene bat colony collapse highlights the importance of hot caves in the Caribbean

**DOI:** 10.1098/rsbl.2024.0700

**Published:** 2025-05-21

**Authors:** J. Angel Soto-Centeno, Reniel Rodríguez Ramos, Pedro Ivo Mônico, Camilo A. Calderón-Acevedo, Justin Bernstein, Lázaro W. Viñola López

**Affiliations:** ^1^ Department of Mammalogy, American Museum of Natural History, New York, NY, USA; ^2^ Departamento de Pedagogía y Ciencias Sociales, Universidad de Puerto Rico, San Juan, Puerto Rico, Puerto Rico; ^3^ Department of Earth and Environmental Sciences, Rutgers University, Newark, NJ, USA; ^4^ Smithsonian Tropical Research Institute, Panama, Panama; ^5^ Department of Biology, University of Florida, Gainesville, FL, USA

**Keywords:** extinction, extirpation, fossil, island, mammal, radiocarbon chronology

## Abstract

Species loss in fragile insular communities can alter the composition and stability of local assemblages. Climate change or anthropogenic pressures are sometimes attributed to the loss of Caribbean bats, but other factors are elusive to document. We studied time-scaled changes in bat assemblage composition from a palaeontological excavation in Cueva Matos, Puerto Rico. Over 800 individual fossils were identified to species, and charcoal was used to develop an AMS ^14^C chronology. Although three bat species live in the cave today, fossils comprise 10 species. These included five extirpated species from the cave and three no longer present on the island. Losses centred around 2460–4470 kya. Notably, we document the first record of *Mormoops megalophylla* as extirpated from Puerto Rico. Nearly 90% of the extirpated bats in Cueva Matos prefer to roost in hot caves where temperatures may reach 40℃. However, these temperatures are currently not held in any cave chamber. Our findings suggest that structural changes in the cave resulted in the loss of heat traps and likely led to a sudden shift in the bat assemblage composition at this cave, which is now void of hot cave specialist bats.

## 1. Introduction

Extinctions influence the composition of biological communities and may alter the ecological and evolutionary processes that shape them. Archipelagos have risen as centres of biodiversity loss, and over 60% of extinctions of terrestrial taxa in the past 1500 years have been island endemics [1]. The Caribbean islands have been on the conservation spotlight because of their combined threats to endemic biodiversity and their habitats [2–4]. Recent studies revealed that about 12% of Caribbean birds have gone extinct since the arrival of humans in these islands [5–7]. More dramatically, this archipelago experienced the loss of over 80% of the flightless terrestrial mammal fauna, most of them since the end of the Pleistocene, around 11−9 kya [6,8,9]. Studies examining Holocene mammal fossils in the Caribbean reflect a strong direct (e.g. hunting) and indirect (e.g. habitat modification) anthropogenic influence, and less so of historical climate change from the end of the Pleistocene [6,8,10–12]. Detailed radiocarbon (^14^C) chronologies have been crucial to refining estimates of species or population loss [13,14]. These chronologically controlled datasets provide an opportunity to quantify the magnitude of species loss and contextualize the possible factors leading to these losses.

Caribbean bats have generally suffered fewer extinctions (i.e. species loss, around 18%) and extirpations (i.e. population loss, around 33%) [8]. Given this lower rate of loss, fossils of Caribbean bats can illuminate patterns of community change over time. Across the Caribbean, about 66% of bats use caves as primary roosts, and roughly half of those, including endemic mormoopid, natalid and phyllostomid bats, exclusively use and have physiological adaptations to roost in hot caves [15–17]. These hot caves house bat demes that may reach hundreds of thousands of individuals. The body heat generated by bats combined with the decomposing guano and the complex cave geomorphology (i.e. chambers with a small and low entrance and high ceiling) creates heat traps with temperatures ranging from 20℃ to 40℃ and up to 99% relative humidity [15]. The stable microclimatic conditions of hot caves allow for non-random aggregations of multiple endemic bat species in the Caribbean [18]. This stability, however, can be jeopardized by natural events. For example, mass mortalities of bats have been reported following major storms in Puerto Rico [19]. A rapid reduction of bat population size can result in the loss of metabolic heat input from the bats and lower the accumulation of guano. This leads to a cascade effect, changing the hot cave microclimate and affecting the dynamics of the bat assembly. Although less studied, changes in cave geomorphology over time, either natural or anthropogenic, also may lead to the loss of heat traps and preclude recolonization by bats, thus reshaping the species composition that uses these roosts. Therefore, hot caves are roosts of conservation importance because they host unique bat assemblages that are highly adapted to and exclusively rely on these fragile conditions.

Puerto Rico, the easternmost island of the Greater Antilles in the Caribbean, has a rich mammal palaeontological history that spans over a century of research. Excavations in caves across Puerto Rico have recovered a wide diversity of extinct mammals, such as sloths (*Acratocnus*), rodents (e.g. *Elasmodontomys*, *Heteropsomys* and *Isolobodon*), and insectivores (*Nesophontes*) [20–22]. Although many fossil bats have been examined, extinct bats reported from excavations in Puerto Rico typically show species inventories without chronological context [23–27]. Here, we provide a chronology for a rich fossil bat assemblage from Cueva Matos in Puerto Rico that resembles a hot cave bat community. We hypothesized that the fossil bat fauna of Cueva Matos was from the middle-to-late Holocene, specifically <6 kya, like other vertebrate faunas of the Caribbean [6,8,28]. We assessed the roosting ecology of each species found as fossils to determine their affinities to hot cave dwelling and compared it with the modern community roosting in the cave today. The diversity presented herein revealed new species records and numerous fossils that inform bat assemblage change over time and modelling of true extinction times.

## 2. Methods

### (a) Cueva Matos cave site

**Figure 1.**
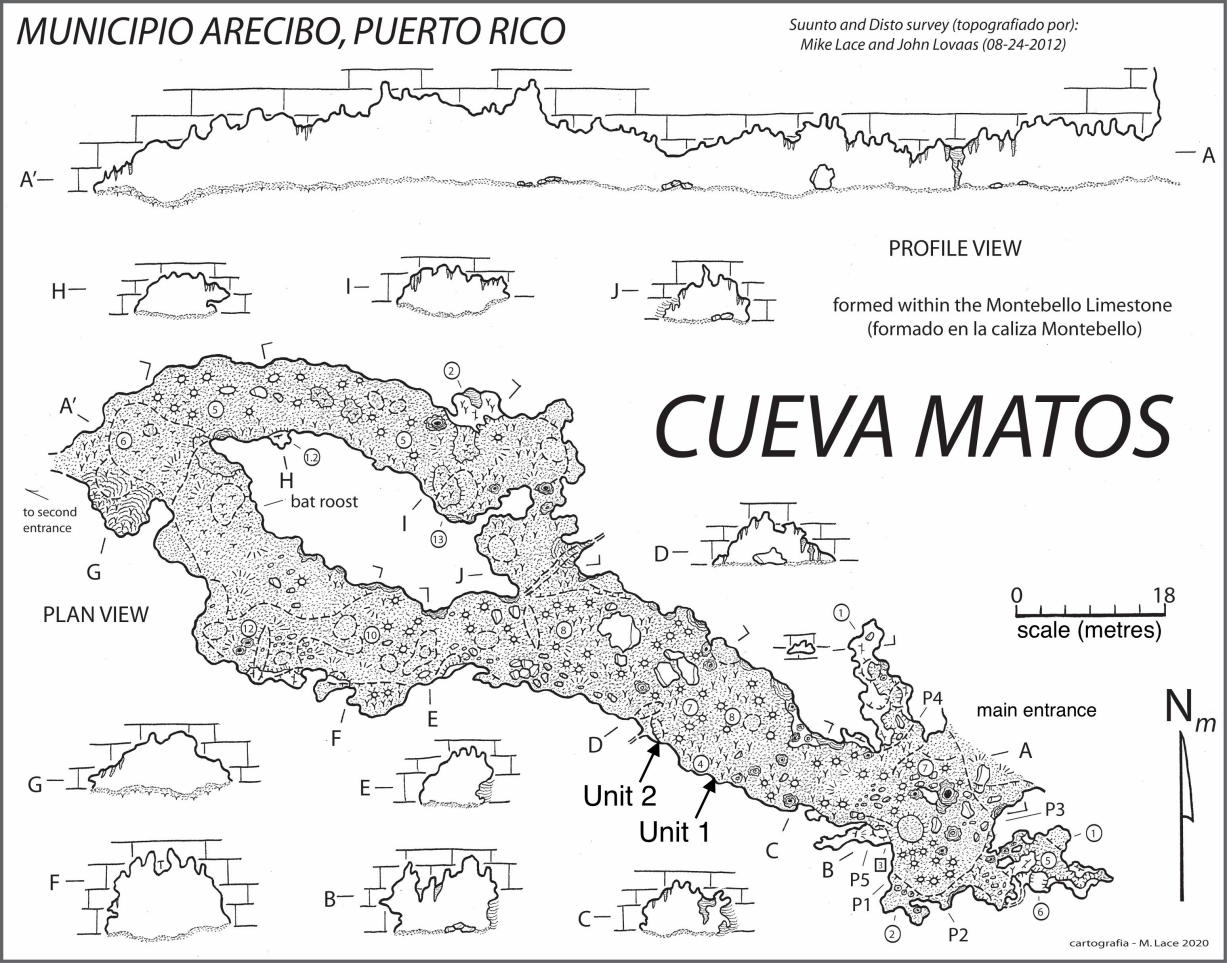
Survey map of Cueva Matos, Arecibo, Puerto Rico. Details show the main entrance to the right, and two localities where excavation units 1 and 2 were performed along the southwest wall of the main gallery. Living bats occupy the darker part of the cave (on the left side), indicated as ‘bat roost’. The survey was performed by Michael Lace and John Lovaas, and cartography was by Michael Lace.

Cueva Matos is in the Barrio Carreras in Arecibo, Puerto Rico (N 18.3, W 66.6; figure 1). This cave is about 10 km inland from the coast in the Cibao Formation. The 220 m long cave boasts a main gallery that is 35 m wide. This cave contains evidence of human presence, including rock art from the latest phases of indigenous occupation of the island and more recent signs of extraction of guano by humans [29]. We took a single-point temperature and humidity reading in the centre of the main chamber using a Kestrel 3500 weather meter to document the current microclimatic conditions of this chamber. Two excavations were performed in the main gallery along the southwest wall of the cave where soil appeared undisturbed (figure 1). Surface fossil material was collected from unit 1, whereas unit 2 consisted of surface material and an excavation of 30 cm long, 30 cm wide and 40 cm deep, dug in artificial layers of 5 cm. Sediment was sieved, and fossils were collected from each layer. We identified all diagnostic fossils to species via direct comparison with modern bones (e.g. dentaries, humeri and skulls) from the American Museum of Natural History collections and a reference collection of JAS-C. The overall minimum number of individuals (MNI) for each species was estimated based on the number of unique bones of the most abundant element (e.g. right humerus) assigned to a species. This study was conducted under a Departamento de Recursos Naturales y Ambientales de Puerto Rico permit (2022-IC-034) to J.A.S.-C.

### (b) Radiocarbon chronology and extinction estimates

Radiocarbon (^14^C) chronologies determine the timing of faunal changes and can be associated to specific historical events [28]. Fossils excavated from unit 2 in Cueva Matos were dark in coloration and lacked collagen needed for direct radiocarbon analysis. Thus, we obtained eight accelerator mass spectrometer (AMS) ^14^C dates from pieces of charcoal (0.01−0.07 g) that corresponded to the artificial layers excavated. Charcoal samples were selected from sediment in each of the individual 5 cm layers, collected using forceps, wrapped in aluminium foil and placed in individual plastic zipper bags. Additionally, a single humerus from the extirpated bat *Monophyllus plethodon* collected from unit 1 in Cueva Matos was used to obtain purified collagen for a direct ^14^C date. All AMS ^14^C dates were used to produce a chronology and estimate the earliest and last appearance dates (EAD and LAD) of the bat fossils from Cueva Matos. These nine ^14^C dates were reported in calendrically calibrated years before present (Cal BP), which represent 95% (2*δ*) estimates. All AMS laboratory analyses were performed at Beta Analytic, Inc., Miami, FL. Specific details of laboratory and calibration methods can be found at https://www.radiocarbon.com/.

Estimates of extinction times for one extirpated and one extinct bat species were performed using the calibration-resampled inverse-weighted McInerny method (CRIWM) [30]. This method accounts for the distributional properties of ^14^C date errors and avoids the assumption that ^14^C dates are normally distributed [31]. CRIWM was implemented in the R package *Rextnct* using a total of 10 Cal BP dates (i.e. five dates per species) [30,32]. Finally, we compiled the individual fossil species data and MNI from unit 2 and examined them across the different excavated levels to determine whether a distinct faunal horizon was present. All raw data and scripts to run the CRIWM analyses are freely available from the Zenodo repository [33].

## 3. Results

Extant bats have been studied extensively across Puerto Rico [34], with a diversity of 13 living species reported on the island [34,35]. Of the total extant diversity, we only documented three species, *Artibeus jamaicensis*, *Brachyphylla cavernarum* and *Erophylla bombifrons*, forming the living deme of Cueva Matos. Previous palaeontological studies have documented the existence of three bat species no longer present in Puerto Rico, the two extirpated species *Macrotus waterhousii* and *Mon. plethodon* and the extinct *Phyllonycteris major*, all in the family Phyllostomidae [23,24].

**Figure 2.**
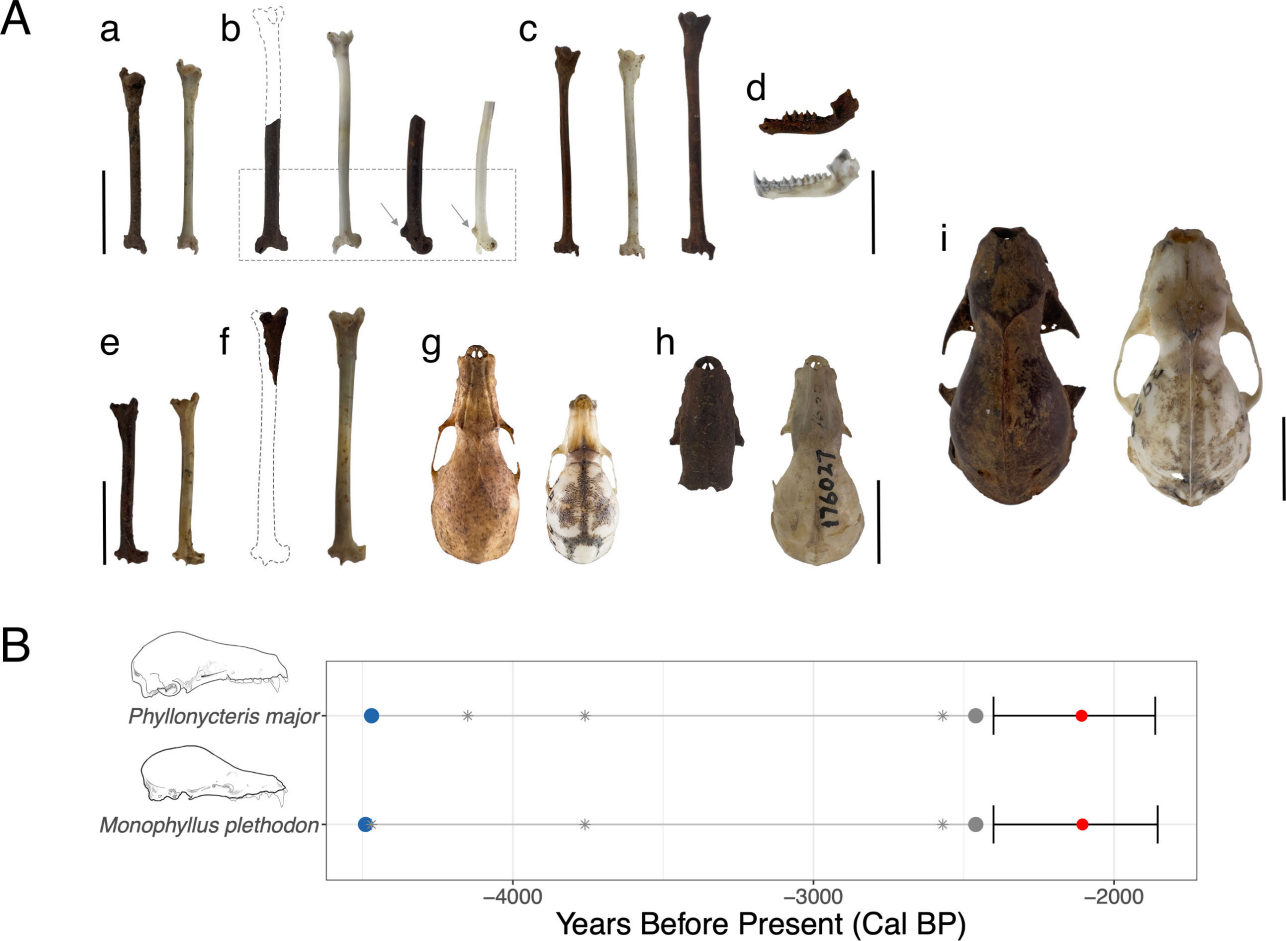
Bat fossil species diversity and estimates of extinction times for Cueva Matos, Arecibo, Puerto Rico. Panel A shows representative extirpated and extinct bats. All scale bars indicate 10 mm. Dotted outlines are shown as a visualization for fragmentary material. In b, the dotted box and arrows indicate details of phenotypic features. From top left: a = *T. brasiliensis* (fossil left humerus, reference right humerus mirror image); b *Mor. megalophylla* (fragment fossil right humerus, right humerus from *Mor. blainvillei* used as reference, lateral view of both humeri of Mormoops with arrows indicating size differences in posterolateral crest); c = *Pte. quadridens* (fossil left humerus, reference right humerus mirror image in centre) and *Pte. portoricensis* (fossil left humerus); d = *Mor. blainvillei* (fossil left dentary above, reference left dentary below); e = *M. redmani* (fossil right humerus, reference left humerus mirror image); f = *E. bombifrons* (fossil right humerus fragment, reference left humerus mirror image); g = *M. plethodon* (fossil cranium, *M. redmani* cranium used as reference); h = *Phy. major* (fossil cranium fragment, *Phy. poeyi* AMNH 176027 used as reference); i = *B. cavernarum* (fossil cranium, *B. cavernarum* AMNH 39295 used as reference). All comparative modern material from J.A.S.-C. reference collection unless noted. Panel B shows estimates of extinction of two bats (*M. plethodon* and *Phy. major*) from Puerto Rico based on calibrated AMS radiocarbon (^14^C) dates. Representative crania for each species are shown to scale as a visual aid. Blue circles indicate the earliest appearance in the fossil record, grey circles represent the LAD, and stars indicate additional radiocarbon dates used to estimate the time of extinction for each taxon. The mean timing of extinction (red circles) and confidence intervals were estimated using CRIWM [30].

The reading of microclimatic conditions by the excavation sites on the main chamber of Cueva Matos indicated the conditions were 25°C and 82% relative humidity, which are characteristic features of cool caves [15]. Fossils excavated from unit 1 included remains of four species of bat, as well as native rodents, amphibians and reptiles. In unit 2, fossils were almost exclusively from bats, except for a few vertebrae of the Puerto Rican boa (*Chilabothrus inornatus*). A total of 2020 bat fossil bones were recovered from Cueva Matos, of which 40% were identifiable to species, and the rest were small fragments. Surface material from unit 1 consisted of 258 fossils of four bat species (table 1). The excavation of unit 2 produced 1762 fossil bones of 10 bat species (table 1, figure 2A). This high diversity includes seven of the 13 species (54%) still extant on Puerto Rico, and three additional species that are no longer present on the island (table 1). Of the three species of bat still extant in Cueva Matos, we recovered fossils of *B. cavernarum* and *E. bombifrons* (figure 2F,I), but no fossils of *A. jamaicensis* were present. Five of the seven extant taxa identified as fossils (71%) are no longer residents of Cueva Matos (table 1). Except for *Tadarida brasiliensis* (figure 2A), the remaining bats extirpated from the cave are considered hot cave specialists (table 1). These hot cave bats included Caribbean endemics (i.e. *Mormoops blainvillei*, *Pteronotus quadridens* and *Mon. redmani*; figure 2C–E) and local endemics (i.e. *Pte. portoricensis*; figure 2C). Notably, we report here the first fossil record of *Mor. megalophylla* for Puerto Rico based on a single partial humerus (table 1, figure 2B). This humerus was characteristically more robust than in the extant *Mor. blainvillei*, and it showed a rounder posterior extension of the trochlea and a noticeably larger posterolateral crest typical of *Mor. megalophylla*.

**Table 1.** Bat palaeo-community of Cueva Matos, Arecibo, Puerto Rico. Taxa are organized in alphabetical order by family. Species known to use hot caves are identified with an asterisk. Elements represents the total number of fossil elements identifiable to a species, and MNI represents the minimum number of individuals for that species (§2). Deposit refers to whether the species was found in unit 1 (U1) and/or unit 2 (U2) in Cueva Matos. Status indicates whether the species is extirpated from the cave (CE) but living elsewhere in Puerto Rico, still living in the cave (L), regionally extirpated (RE) but living in other Caribbean islands or the mainland, and extinct (E). The LAD is presented as the radiocarbon age in years BP.

taxon	elements (MNI)	deposit	status	conventional ^14^C age (yr BP) range	sample no.
Molossidae					
*T. brasiliensis*	1 (1)	U2	CE	4150 ± 30	Beta−639851
Mormoopidae					
* Mor. blainvillei	6 (6)	U2	CE	4470 ± 30 to 2460 ± 30	Beta−6 39 849
* Mor. megalophylla	1 (1)	U2	RE	3760 ± 30	Beta−6 39 850
* Pte. portoricensis	94 (29)	U1, U2	CE	4470 ± 30 to 2460 ± 30	Beta−6 39 852 (U2)
* Pte. quadridens	82 (30)	U2	CE	4470 ± 30 to 2460 ± 30	Beta−6 39 849
Phyllostomidae					
*B. cavernarum*	286 (86)	U1, U2	L	4490 ± 30 to 660 ± 30	Beta−6 39 848 (U2)
* E. bombifrons	3 (3)	U2	L	4470 ± 30 to 2570 ± 30	Beta−6 39 853
* Phy. major	124 (36)	U2	E	4470 ± 30 to 2460 ± 30	Beta−6 39 849
* Mon. plethodon	32 (13)	U1, U2	RE	4490 ± 30 to 2460 ± 30	Beta−6 39 849 (U2)
* Mon. redmani	25 (14)	U1, U2	CE	4490 ± 30 to 2570 ± 30	Beta−6 39 853 (U2)

Two phyllostomid bats, the extirpated *M. plethodon* (figure 2G) and the extinct *Phy. major* (figure 2H), have been previously reported from other fossil deposits on the island [23,24], although at a lower density as represented in Cueva Matos. Given their relationship to other hot cave bats (i.e. *M. redmani* and *Phy. poeyi*) and their presence along other hot cave bat species, both *Mon. plethodon* and *Phy. major* likely used hot caves in Puerto Rico. Overall, 32 elements of *Mon. plethodon* and 124 elements of *Phy. major* including crania, dentaries and humeri for both species were recovered. These represent a minimum number of individuals (MNIs) of 13 and 36 for each species, respectively. Estimates based on CRIWM from five ^14^C dates, each of *Mon. plethodon* and *Phy. major* show that these taxa had median extinction dates of 2105–2108 cal. years BP (95% CI = 1855−2401; figure 2B).

**Figure 3.**
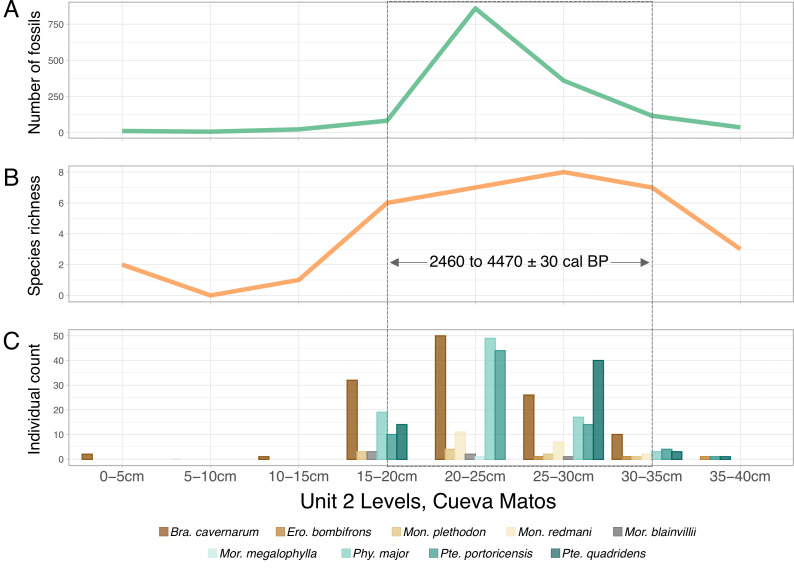
Distribution of bat fossil diversity in Cueva Matos, Arecibo, Puerto Rico. Faunal horizon plots of artificial layers in unit 2 indicating abundance in number of fossils (panel A), species richness (panel B) and individual species (panel C). Plots show a peak in abundance data in the 20−25 cm layer, a peak in species richness at 25−30 cm and a distribution of highest species diversity from 15 to 35 cm.

Fossil abundance and species richness quantified at each level of unit 2 indicated a single faunal horizon (figure 3). We estimated a total of at least 219 individuals identified from our excavation (table 1). Abundance data showed a distinct peak spanning 20−25 cm (figure 3A), whereas species richness peaked at 25−30 cm and decreased steadily with depth (figure 3B). The highest number of species was observed between 15 and 35 cm (figure 3C). Peak abundance and species richness span over two millennia and ranged from 2460 to 4470 (±30 cal. BP). The ^14^C dates and CRIWM extinction estimate results support our hypothesis that the bat colony collapse of Cueva Matos is younger than 6 ky, which is important to help isolate the possible factors contributing to species loss from a community.

## 4. Discussion

The top modern threats to Caribbean bats all relate to human-driven factors, such as habitat loss, climate change, disturbance and invasive species [36,37]. Bat extinctions and extirpations from across the Caribbean have been attributed to recent phenomena with a strong anthropogenic influence [6,8,9], but other forces driving these losses, such as changes in cave geomorphology, are often unexplored. Studies show that Caribbean bat assemblages of the past may include multiple species that are not present in the living fauna [28,38,39]. Notably, about 75% of the bat extinction events reported in the Caribbean involve cave-dwelling species [39]. Although that number drops to about 25% when considering Puerto Rico alone, it is still a significant proportion. Herein, we studied the fossil community assemblage of Cueva Matos in Arecibo, Puerto Rico. The fossil bats from this cave are characteristic of a hot cave bat assemblage, and we aimed to provide a chronological context to determine the timing of population and species loss for this site.

The fossil bat species assemblage in Cueva Matos includes members of 60% of the bat families existing on the island (i.e. Molossidae, Mormoopidae and Phyllostomidae). An extant species in the cave, *E. bombifrons,* was represented by a single partial humerus. The also extant *B. cavernarum* was the most abundant species in the fossil samples and appeared as recently as 660 ± 30 cal. BP. The estimated number of individuals (i.e. MNI; table 1) found as fossils for the living species of Cueva Matos may be indicative of their relative abundance in the cave over time and the length of time that they have used this roost. Furthermore, the overall estimated MNIs (= 219) represent a large density given the small size of the excavation (about 0.036 cm^3^). This suggests that the bat colony in this gallery of Cueva Matos likely consisted of thousands of individuals, perhaps similar in size to other hot caves in the Caribbean.

The diversity lost included bat species that represent most ecological guilds existing on the island, such as hawking insectivores, understory insectivores, frugivores and nectarivores [35]. Remarkably, this diversity of ecological guilds was drastically reduced by the local extirpation of mormoopid bats (i.e. *Mormoops blainvillei*, *Mor. megalophylla*, *Pte. portoricensis* and *Pte. quadridens*) from this roost around 2460 ky. Two lost species, *M. plethodon* and *Phy. major*, were previously described from five and 60 fragmentary skulls, respectively, from the type locality in Cueva Catedral in Morovis [23,40]. The presence of these bats in the Cueva Matos deposit denotes a range extension of 30 km west of Cueva Catedral. Both *Mon. plethodon* and *Phy. major* were present in layers ranging from 15 to 35 cm depth and associated with fossils of other phyllostomid (i.e. *Mon. redmani*) and mormoopid bats (i.e. *Mor. blainvillei*, *Pte. portoricensis* and *Pte. quadridens*) that are known to roost in hot caves [15,41].

The estimated MNI of *Mon. plethodon* and *Phy. major* in Cueva Matos was higher than that of *Mon. redmani* and *E. bombifrons*, which both are extant on the island, have similar ecological affinities (i.e. hot cave dwelling and mainly nectarivorous and frugivorous diets) and are closely related to the extinct species. Accounts of fossils in caves from Cuba show that both *Mon. redmani* and *E. bombifrons* appear to be less abundant in recent times than in the past [42]. More sampling from Cueva Matos is needed to understand if our findings about population density represent an artefact of bias in fossil preservation, natural fluctuations in population size, differential roost selection preferences or an indication that the larger *Mon. plethodon* and *Phy. major* could be dominant in that cave deme. However, the ubiquitousness of *Mon. plethodon* and *Phy. major* allowed us to model true extinction estimates of about 2100 cal. years BP. These estimates revealed that both bats were resilient to drastic changes in island area due to sea-level rise at the end of the Pleistocene and survived for about 2–3 millennia following the human colonization known to correlate with the loss of regional biodiversity [5,6,8,9,43]. The late Holocene loss of these bats matches the timing of extinction of mormoopid, natalid and phyllostomid bats in other Caribbean islands [6,8,14,44] and highlights a loss of phylogenetic diversity for Puerto Rico. The time span of occurrence, consistent association with other species and number of individuals documented suggest that the extinct *Mon. plethodon* and *Phy. major* were likely stable members of the community of Cueva Matos.

We also documented the first record of *Mor. megalophylla* on Puerto Rico, which dated to 3760 ky (± 30 cal. BP), and contributes to a broader understanding of the late Holocene extirpation of this taxon [13,27]. This bat became regionally extirpated from many Caribbean islands (i.e. The Bahamas, Cuba, Hispaniola and Jamaica, Marie-Galante and Barbuda in the Lesser Antilles and Curaçao and Margarita off the coast of Venezuela) [27]. Nonetheless, this bat still lives on the mainland, from southern Texas in the United States to northwestern South America and through Trinidad and Tobago [13,27,38,42,45]. The fossil record presented herein closes a biogeographic gap spanning the Greater and Lesser Antilles. The two closest records for this extinct bat in the Caribbean are about 530 km to the west in Cerro de San Francisco, Dominican Republic and about 415 km to the east in Barbuda, Lesser Antilles. Caribbean fossil records of *Mor. megalophylla* revealed its presence alongside other mormoopid bats known to roost in hot caves. The single partial distal humerus we present herein suggests that *Mor. megalophylla* may have existed in low densities on Cueva Matos alongside all other mormoopid bats from Puerto Rico. We expect that further palaeontological work leveraging chronologically controlled excavations on Puerto Rico will likely produce similar extinct or extirpated species records for the island.

Between 71% and 86% of the species found as fossils across all layers examined were from bats that exclusively roost in hot caves [15,41]. These included the locally extirpated *Mor. blainvillei*, *Pte. portoricensis*, *Pte. quadridens* and *Mon. redmani*, the regionally extirpated *Mon. plethodon* and *Mor. megalophylla*, and the extinct *Phy. major*. Fossils of two species, *E. bombifrons* and *B. cavernarum*, still extant in Cueva Matos, were present in the upper layers from 0 to 25 cm. Both species typically roost in cool to warm chambers of caves where temperatures typically do not exceed 27°C [15,46]. Their presence in this community highlights their resilience over time allowing them to persist in the cooler parts of Cueva Matos today and indicating that they may have used this main gallery as a roost in the recent past.

Caves in the Caribbean are abundant, especially in karst rock formations, and provide multiple advantages to bat communities across this archipelago. Given their specific geomorphology, hot caves are less numerous [46,47]. Threats to hot caves are deemed as potentially key drivers of extinction for bats in the Caribbean, largely because of the uniqueness of this roost type, and the large proportion of endemic species that use such caves [38,48,49]. Caribbean hot caves may host colonies reaching tens to hundreds of thousands of individual bats that are physiologically adapted to exclusively use this type of roost [15,41]. Large hot cave bat colonies may undergo natural fluctuations in numbers of individuals and species packing following environmental phenomena, such as hurricanes [19]. Furthermore, studies suggest that guano harvesting may contribute to colony disturbance as it effectively removes one of the sources that contribute to heat generation in hot caves [47,48]. Perhaps because of the difficulty in measuring its impact or because cave roosts are considered stable over millennia, changes in cave geomorphology are rarely included as a threat. Despite that nearly all species of bat identified as extirpated or extinct have hot cave affinities, our measurements of the cave microclimate show that this roost does not sustain a hot environment suitable for these bats. Furthermore, the main gallery of Cueva Matos has a significantly large entrance, inconsistent with the geomorphological characteristics of typical hot caves, which are small and low to the ground. Combined, the data we presented indicate a drastic change in the bat assembly of Cueva Matos. This loss of species could likely be linked to a rapid change in the cave’s microclimate, which drove bats away from this roost and precluded recolonization. Thus, this diverse bat deposit likely represents the species assemblage at a time when the main gallery was completely or nearly closed, allowing it to retain high temperatures that promoted the occurrence of hot cave bats. It is likely that the collapse of the wall of the main gallery in Cueva Matos, now considered the main cave entrance, resulted in the loss of the hot cave microclimate and the observed collapse of the bat colony. These types of disturbance have only been posited as potential factors for species loss, but have been elusive to document. The extinction and extirpation bias towards hot cave-dwelling bats in Cueva Matos highlights the importance of this unique resource for maintaining local species diversity.

## Data Availability

Raw data of all fossils collected, including unidentifiable fragments, and R code and data to reproduce CRWIM analysis are deposited and freely accessible from the Zenodo repository [33].
